# A randomized controlled trial to pilot the efficacy of a computer-based intervention with elements of virtual reality and limited therapist assistance for the treatment of post-traumatic stress disorder

**DOI:** 10.3389/fdgth.2022.974668

**Published:** 2022-10-18

**Authors:** Marieke van Meggelen, Nexhmedin Morina, Colin van der Heiden, Willem-Paul Brinkman, Iris E. Yocarini, Myrthe L. Tielman, Jan Rodenburg, Elisa van Ee, Kevin van Schie, Marijke E. Broekman, Ingmar H. A. Franken

**Affiliations:** ^1^ Department of Psychology, Child and Education Studies, Erasmus School of Social and Behavioural Sciences, Erasmus University Rotterdam, Rotterdam, Netherlands; ^2^ Parnassia Group, Outpatient Treatment Center PsyQ, The Hague, Netherlands; ^3^ Department of Clinical Psychology and Psychotherapy, University of Münster, Münster, Germany; ^4^ Parnassia Group, Outpatient Treatment Center PsyQ, Rotterdam, Netherlands; ^5^ Department of Intelligent Systems, Delft University of Technology, Delft, Netherlands; ^6^ Leiden Institute of Advanced Computer Science, Leiden University, Leiden, Netherlands; ^7^ De Hemisfeer, Praktijk Voor Psychotrauma / Migratieproblematiek, ‘s-Hertogenbosch, Netherlands; ^8^ Reinier van Arkel, Psychotrauma Centrum Zuid-Nederland, ‘s-Hertogenbosch, Netherlands; ^9^ Behavioural Science Institute, Radboud University, Nijmegen, Netherlands; ^10^ MRC Cognition and Brain Sciences Unit, University of Cambridge, Cambridge, United Kingdom

**Keywords:** post-traumatic stress disorder, PTSD, intervention, computer-based, virtual reality, war veterans, childhood sexual abuse

## Abstract

Although well-established therapies exist for post-traumatic stress disorder (PTSD), barriers to seek mental health care are high. Technology-based interventions may play a role in improving the reach of efforts to treat, especially when therapist availability is low. The goal of the current randomized controlled trial was to pilot the efficacy of a computer-based trauma intervention with elements of virtual reality (VR; *3MR* system) and limited therapist involvement for the treatment of PTSD in a childhood sexual abuse (CSA) and war veteran sample and to compare this to “treatment as usual” (TAU). TAU consisted of evidence-based approaches such as imaginal exposure, EMDR, or narrative exposure therapy. A total of 44 patients with PTSD were included and randomly assigned to 12 sessions of *3MR* intervention or TAU (completer *n 3MR* = 12, TAU = 18). Several measures (PCL-5, BDI-II, OQ-45-2, and the M.I.N.I. 5.0.0.) were administered to measure symptoms of PTSD and depression and scores of overall well-being at pre, post, and a three-month follow-up measurement. Analyses suggest that symptoms of PTSD and depression in the *3MR* condition decreased, and overall well-being increased between pre and post measurements. Results did not indicate any clear differences between the treatment conditions over time which suggests that treatment gains of the *3MR* intervention seem no less than those of TAU. Finally, both treatment conditions produced similar remission rates of PTSD and depression. Therefore, the *3MR* intervention could possibly constitute an appropriate treatment alternative. The small sample size as well as evident drop-out rates in the *3MR* condition (45%) do warrant further research. The procedures of this study were approved by the Medical Ethical Research Committee (MERC) of the Erasmus Medical Center in Rotterdam (MEC-NL46279.078.13) and pre-registered *via* ClinicalTrials.gov (Protocol Record CI1-12-S028-1).

## Key practitioner message

• The *Multi Modal Memory Restructuring* (*3MR*) system is a computer-based trauma intervention with elements of virtual reality (VR) and limited therapist involvement for PTSD.
• We conducted a randomized controlled trial to pilot the efficacy of the 3*MR* intervention, compared to “treatment as usual” (TAU).
• Patients were survivors of childhood sexual abuse and war veterans.
• Findings indicate that the *3MR* intervention decreased PTSD and depression symptoms between pre, post and follow-up measurements.
• There were no clear differences found between the two treatment conditions which suggests that the treatment gains of the *3MR* intervention seem no less than those of TAU. However, the small sample size and the evident drop-out rate warrant further research.

## Introduction

Although many people experience traumatic events during their life, only a small minority (approximately 5.6% – 7.8%) develops post-traumatic stress disorder (PTSD) afterwards (1–3). Estimated prevalence rates vary across high and low income countries, as well as between trauma types (2, 4, 5). Physical violence is associated with significant population burden of PTSD (i.e., the associated number of years of PTSD per trauma type on a population level) and predicts subsequent physical violence and intimate partner sexual violence (4). Furthermore, war-related traumatic events are associated with a more chronic course of PTSD symptom recovery relative to exposure to other traumatic events, showing the longest median duration (5 years) of PTSD symptoms (4). In general, the majority of individuals with PTSD fail to recover even after many years and mean symptom duration is significantly longer than previously thought (6, 4). The DSM-5 (Diagnostic and Statistical Manual of Mental Disorders 5th Edition) clusters PTSD symptoms in the categories re-experiencing, avoidance, negative alterations in mood and cognition, and alterations in arousal and reactivity (7). People with PTSD for example re-experience the traumatic event unwantedly by means of memory flashbacks or nightmares, avoid stimuli associated with the traumatic event and experience numbing of general responsiveness and symptoms of increased arousal. PTSD symptoms often chronically impair peoples’ daily life, but nevertheless barriers to seek mental health care seem high. Researchers found in a military sample (*n* = 6,000) that only 23%–40% of respondents who met criteria for a mental disorder sought professional help (8). Similar, it was reported that only 18.9% of rape victims in a National Women’s Study (*n* = 4,006) sought formal or informal help for their PTSD or major depressive symptoms (9). Reasons for peoples’ reluctance in seeking help might relate to fear of stigmatization, fear to be seen as weak or to be treated differently (8, 10). In addition, there exist different structural barriers to health care (11). An example is that of waiting lists. Waiting times of several months are commonplace and have resulted in numerous reports warranting change (e.g., 12, 13). Although widely examined and approved therapies for PTSD, such as Eye Movement Desensitization Reprocessing (EMDR) and (trauma-focused) cognitive behavioral therapy [(CBT), 14–16] exist, these current standards of at least 8 to 12 weekly sessions with a therapist lasting for 60 to 90 min can be seen as intensive and therefore costly, in particular in low and middle income countries. In a world that is changing, and where factors as confined access to mental health care due to long travel distances, financial restraints and a limited number of available therapists is current, there remains an ongoing need for interventions that aim to improve the effectiveness, cost-effectiveness, and above all accessibility to treatment for individuals with PTSD.

The current study for the first time describes the efficacy of a novel computer-based trauma intervention with elements of virtual reality (VR) and limited therapist involvement for the treatment of PTSD, offered *via* the *3MR* system. The *3MR* system (17) comprises a software application that focuses on the restructuring and relearning of past events. The *3MR* system allows patients to visualize past events using personal photos, narrative texts, online geographical maps and patient-created 3D virtual worlds that can be viewed on a computer screen. The *3MR* intervention hinges on two fields of research, namely of VR containing patient created 3D virtual worlds and of computer-based interventions enabling patients to follow through therapy sessions in their own environment and without the direct presence of a therapist.

### Virtual reality exposure therapy (VRET)

Exposure to the traumatic memories or cues for the traumatic event often plays an important role in reducing symptoms of PTSD (18). Exposure *in vivo* might often be difficult to establish, mainly due to practical reasons such as high costs (e.g., travelling to other countries). Also, it might be dangerous to return to the original surroundings of the traumatic event (e. g., in case of a warzone). Using VRET to extinct fearful stimuli can overcome these issues since it uses computer-generated environments to simulate feared stimuli (e.g., 19). In these virtual environments, users can be systematically exposed to specific stimuli within a contextually relevant setting, for example a warzone or airplane for soldiers with war-related PTSD (20, 21). Many forms of VR exist, and can be defined as “the use of computer and behavioral interfaces to simulate the behavior of 3D entities that interact in real-time with each other and with a user immersed *via* sensorimotor channels” (22). Experts (23) mention a wide range of applications that fit this definition, from 3D video games to large immersive VR rooms (the so-called CAVEs) and describe that over the last few years, large expensive VR installations have made room for Head Mounted Displays (HMDs) and other more affordable technologies used in mental health care. Recent studies also describe the development of VR over the last decades and the increase of research interest on the use of VR to improve mental health (24, 25). This has led to applications for the treatment of anxiety disorders (e.g. 19), resulting in several studies describing positive treatment outcomes for several disorders (26), including PTSD (e.g., 27–30, 20). In a systematic review, researchers included ten studies that used VRET to conduct exposure in CBT treatment for PTSD and found that VRET is a potentially promising treatment option (31). The researchers also highlight several limitations to this research field (e.g., only few studies available in the literature, no standardized number of therapy sessions and the non-use of intent-to-treat analysis). Similar, in a recent meta-analysis researchers compared ten clinical trials on the efficacy of VRET for the treatment of PTSD. They found that VRET for PTSD significantly outperformed inactive control conditions and did not differ from active control conditions (32).

### Computer-based interventions

Considering the need for accessible treatments, computer-based interventions are known for their ability to reach large groups of people. This type of intervention has several benefits compared to traditional therapy; they are often personalized and tailored to the needs of a diverse group of users, they can reach a large population at relatively low cost and they can be used from a person’s own home (33). Generally, computerized interventions yield comparable effect sizes as traditional psychosocial interventions in the treatment of depression and anxiety (27, 33–36). Differences exist into what extent assistance is offered during these interventions (e.g., no, administrative or therapist assistance; 37). Drop-out rates in computer-based interventions are considered a cause of concern (38). Level of therapist involvement however does seem to influence drop-out (37). Altogether, interventions that require limited therapist involvement (with costs of therapists arguably accounting for the largest proportion when treating patients) and in addition lower expenses such as travel costs, can potentially improve cost-effectiveness and access to mental health care.

The goal of the current randomized controlled trial was to (1) pilot the efficacy of a computer-based trauma intervention with elements of VR (*3MR* system) and limited therapist assistance for the treatment of PTSD in a CSA and war veteran sample; and (2) to compare this to “treatment as usual” (TAU). We therefore tested whether the *3MR* intervention decreased symptoms of PTSD, depression and increased overall well-being at post and follow-up measurements, and if the outcome of the *3MR* intervention was comparable to TAU. It was expected that *3MR* would perform similarly to TAU.

## Method

### Procedure

#### Study enrollment

Patients enrolled for treatment *via* the specialized mental health care centers of PsyQ (locations Rotterdam-Kralingen, Spijkenisse, and The Hague), Reinier van Arkel (Psychotrauma Centrum Zuid-Nederland), and the ambulatory of the Erasmus University Rotterdam (Department of Clinical Psychology). Potential participants that presumably met in- and exclusion criteria were given a detailed information sheet about the project. There were at least five days between the first and second consultation so that potential participants had the chance to (re-)consider whether they truly wanted to participate in this study. If individuals were interested in participation, they filled in an informed consent^1^.

#### In- and exclusion criteria

Following consent, the in- and exclusion criteria were checked in an extensive interview by telephone that was administered by trained psychologists and psychology master students. Assessment of the in- and exclusion criteria included a semi-structured clinical interview [Mini International Neuropsychiatric Interview Plus - Dutch Version 5.0.0 [M.I.N.I. Plus 5.0.0], (39)] and a self-report questionnaire [Dissociative Experiences Scale [DES], (40)]. Participants were excluded if they met criteria for a current bipolar disorder, current psychotic episode, if they were actively suicidal (defined as “high risk” according to the Mini International Neuropsychiatric Interview Plus - Dutch Version 5.0.0 [M.I.N.I. Plus 5.0.0., (39, 41)], or scored a total score of ≥40 on the Dissociative Experiences Scale [DES, (40)]. Co-morbidity as such was not an exclusion criterion, but PTSD had to be the primary diagnosis according to the M.I.N.I. Plus 5.0.0. (Initially, it was agreed to include patients that met criteria for the diagnosis of PTSD and/or Depression [according to the M.I.N.I. 5.0.0.] as primary diagnosis. However, since recruitment took place at the trauma departments of the participating mental health care centers, only one patient did not meet criteria for the diagnosis of PTSD [only for Depression] at initial screening and inclusion. Therefore, the main focus of the article is on PTSD [e.g., background, introduction, discussion]). Use of medication was no exclusion criterion, provided that the dose was stable for at least two weeks at the beginning of the therapy, remained stable throughout therapy, and was closely monitored.

#### Measurements

To determine PTSD and depression symptom levels, overall well-being and to check the criteria for meeting the diagnosis of PTSD and/or depression at pre-, post- and 3-month follow-up measurements, primary and secondary outcome measures (PTSD checklist for the DSM 5 with LEC and extended Criterion A* [PCL-5, (42)]; Beck Depression Inventory – Second Edition [BDI-II, (43)]; Outcome Questionnaire – 45 – Second Edition [OQ-45-2, (44)]) were assessed *via* online self-report questionnaires, as well as the M.I.N.I. Plus 5.0.0. *via* telephone. (The M.I.N.I. Plus 5.0.0. and the PCL-5 are based on the criteria for PTSD set by the DSM IV and DSM-5 respectively, and thus differ. However, in light of the emergence of the Dutch PCL-5 questionnaire during the onset of this study (2013), this was the soundest option to reflect the diagnostic changes in the field of PTSD at that time, with consideration of existent measures. For an overview of differences between DSM IV and DSM-5 we refer to Friedman (45)]. Assessors were independent but not blinded to treatment condition. At 12-months post treatment, another follow-up measurement was conducted, this assessment is ongoing and is not further described in the present study. Participants were asked to preferably not seek other forms of therapy between the post and the 3-month follow-up measurement unless indicated by the therapist. In the control condition, this restriction was not given due to practical reasons.

#### Randomization

This study had a randomized controlled design. The control condition was TAU. Randomization was conducted after inclusion. Eligible patients were randomly assigned to two conditions by an independent researcher *via* a random-numbers table and its allocation sequence was computer-generated. A stratified randomization procedure was employed for clients with CSA or war related PTSD. Patients were assigned to groups by the first author (MM) (i.e., disclosure of assigned therapy condition by opening randomization envelopes set up by the independent researcher), following the assessment of inclusion and exclusion criteria and randomization. Participants were not compensated for their contribution to the study and were free to leave the study at any time and (in the *3MR* condition) receive TAU instead.

### Participants and flow

**Figure 1:**
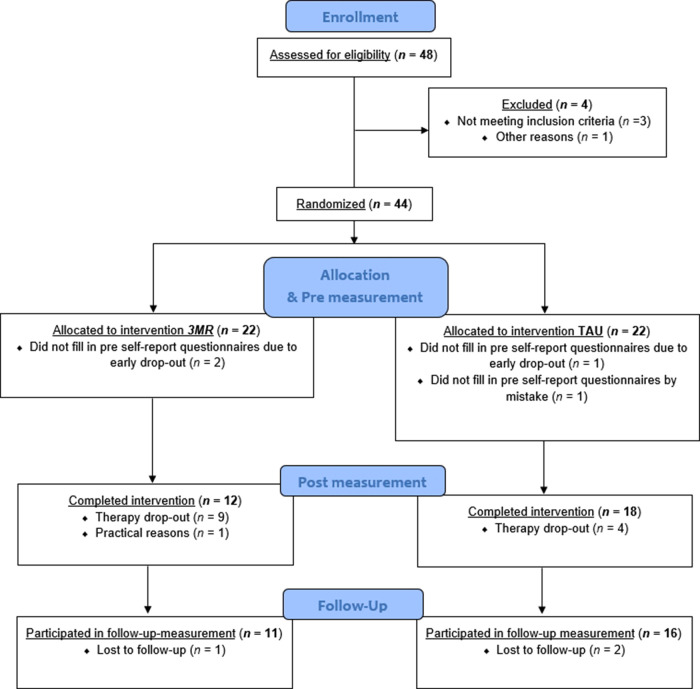
Flow diagram of the study.

The research population consisted of both war veterans and survivors of CSA. Selection of this trauma population had a practical background since these trauma types were most prevalent in the participating mental health care centers. Survivors of CSA could have either single or multiple/recurrent traumatic experiences that had occurred between 0 and 18 years old. Recruited veterans presumably served (a) Dutch military mission(s) in Lebanon, Bosnia-Herzegovina, Iraq, or Afghanistan. Following pre-screening, a total of 83 patients were referred *via* the participating mental health care centers. Of the 83 individuals that were contacted by the researchers, 48 registered interest in the study. Of these 48 individuals, three did not meet in- and exclusion criteria and one could not join the study due to practical reasons (not able to follow therapy sessions at home). See Figure 1 for a flow of the participants through the trial.

Participants were aged between 20 and 62 years, with an average age of 39 years. Forty-eight percent was female, 47.7% had previously followed therapy, and 43.2% took prescription drugs during the study period. See Appendix 1 Table 1 for participant descriptives specified per treatment condition (*3MR* and TAU). Appendix 2 Table 2 shows that independent samples *t*-tests revealed no significant baseline differences between conditions. A total of 14 patients dropped out of the study. See also Figure 1 and section 4.3 Drop-out analysis. Thirteen individuals could be classified as therapy drop-out, whereas one individual dropped out because of practical reasons. Originally, it was planned to recruit a total of 144 participants to meet sufficient power levels. Patient recruitment turned out to be more difficult than anticipated. After many attempts to facilitate the recruitment we had to stop the inclusion of patients after two years because of practical and financial reasons.

**Table 1:** Descriptive statistics of patients in 3MR and TAU conditions.

Condition	*N*	Age^a^ *M (SD)*	Male gender %	Trauma group (CSA or veteran)	Previous therapy %	Medication^b^ %	Pre PCL-5 *M (SD)*	Pre BDI-II *M (SD)*	Pre OQ-45-2 *M (SD)*
*3MR*	22	42.05 (12.15)	50.0%	54.5% CSA 45.5% Veteran	59.1%	48.8%	45.45 (13.41)	25.56 (7.92)	85.10 (25.32)
*TAU*	22	36.55 (10.43)	55.5%	54.5% CSA 45.5% Veteran	40.1%	51.2%	49.10 (10.22)	28.95 (9.37)	86.40 (16.56)

^a^ Missing age and pre measurement PCL-5, BDI-I-II and OQ-45-2 data *n* = 4 (3MR [*n* = 20], TAU [*n* = 20]).

^b^ Missing Medication data *n* = 1 (3MR [*n* = 21], TAU [*n* = 22]). CSA, Childhood Sexual Abuse; PCL-5, PTSD Checklist for the DSM 5; BDI-II-NL, Beck Depression Inventory – Second Edition; OQ-45-2, Outcome Questionnaire – 45 – Second Edition; 3MR, Multi Modal Memory Restructuring; TAU, Treatment as Usual.

**Table 2:** Independent samples t-test baseline (pre) differences between 3MR and TAU condition.

	*N*		t	df	*p*
	3MR	TAU			
Age^a^	20	20	−1.536	38	0.133
Gender	22	22	−0.295	42	0.769
Trauma group	22	22	0.000	42	1.000
Previous therapy	22	22	−1.198	42	0.238
Medication^b^	21	22	0.167	41	0.868
PCL-5	20	20	0.968	38	0.399
BDI-II	*20*	20	1.240	38	0.223
OQ-45-2	20	20	0.192	38	0.849

^a^ Missing age and pre measurement PCL-5, BDI-I-II and OQ-45-2 data *n* = 4.

^b^ Missing Medication data *n* = 1. 3MR = Multi Modal Memory Restructuring; TAU, Treatment as Usual; PCL-5, PTSD Checklist for the DSM 5; BDI-II-NL, Beck Depression Inventory - Second Edition; OQ-45-2, Outcome Questionnaire – 45 – Second Edition.

### Materials

#### Semi-structured clinical interview

To assess primary diagnosis of PTSD and/or depression, the Dutch version of the *M.I.N.I. Plus 5.0.0* was administered (M.I.N.I. Plus 5.0.0. (39), M.I.N.I. Plus 5.0.0 NL [i.e., the Dutch version for use in the Netherlands (41)]. The M.I.N.I. Plus 5.0.0. is a structured clinical interview used to assess Axis I Disorders according to DSM IV. All questions are yes/no questions and based on the answers, it is determined whether the patient meets the criteria for a certain disorder (e.g., “In the past month, have you avoided thinking about the event, or have you avoided things that remind you of the event?”). The M.I.N.I. 5.0.0. has excellent interrater reliability (*κ *> 0.75), very good test-retest reliability (*κ *> 0.75), as well as validity (39).

#### Self-report questionnaires

 The *Dissociative Experiences Scale* [DES (40)] was used to measure dissociative experiences that the participant may be suffering from due to PTSD. The DES consists of 28 self-report items rated on a scale of 0 to 100. Subjects indicate to what extent they experience certain symptoms such as amnesia. An example item is “Some people have the experience of finding themselves in a place and having no idea how they got there. Select a number to show what percentage of the time this happens to you.” The DES has good test-retest reliability (*r* = 0.84) and split-half reliability (*r* = 0.71–0.96), as well as good internal consistency and construct validity (*ρ* = 0.64) (40).

Self-reported symptoms of PTSD were assessed using the *PTSD checklist for the DSM 5* with LEC and extended Criterion A [PCL-5 (42, 46)]. The PCL-5 is a 20-item self-report measure assessing the symptoms of PTSD according to the DSM 5. An example item is “In the past month, how much were you bothered by repeated, disturbing, and unwanted memories of the stressful experience?” on a scale of 0 (*not at all*) to 4 (*extremely*). The PCL-5 was found to have strong internal consistency (*α* = 0.94), test-retest reliability (*r* = 0.82), and convergent (*rs* = 0.74 to 0.85) and discriminant (*rs* = 0.31 to 0.60) validity (Blevins et al., 2015).

The *Beck Depression Inventory – Second Edition* (BDI-II (43); BDI-II-NL [Dutch version (47)] is a 21-item self-report instrument that assesses severity of depression. Each item has four options ranging from 0 (“not present”) to 3 (“present all the time”) within the previous two weeks. Higher scores indicate a more severe depression. An example item is “I don’t feel I am worse than anybody else - I am critical of myself for my weaknesses or mistakes - I blame myself all the time for my faults - I blame myself for everything bad that happens”. The BDI-II has shown good internal consistency reliability [*α* = 0.93 amongst college students, *α* = 0.92 amongst outpatients, (43)].

Overall well-being was assessed using the *Outcome Questionnaire – 45 – Second Edition* [OQ-45–2, (44)]. It measures symptom distress (SD), interpersonal relationships (IR) and difficulties in social roles (SR). The participant indicates how often the statements applied to them in the past week on a scale of 0 (“never”) to 4 (“almost all the time”). An example item is “I have difficulty concentrating”. It has been shown to have good test-retest reliability (*r* = 0.79), good internal consistency reliability (*r* = 0.92) as well as good concurrent validity (*α* = 0.68–0.80) in Dutch clinical populations (48).

#### Apparatus

The *Multi Modal Memory Restructuring* (*3MR*) system is a software application, which focuses on the restructuring and relearning of past events and can be operated by the patient without or with minimal therapist assistance. The *3MR* system allows people to visualize past events using personal photos, narrative texts, online geographical maps, and patient-created 3D virtual worlds. Patients view the *3MR* application on their computer screen. In this trial there was no use of Head Mounted Displays. The *3MR* system consists of a period overview and a diary, which contains the tools “Text”, “Images”, “Website”, “Media”, “Webcam” and “3D world”. Patients install the *3MR* system on their own PC or laptop. The *3MR* system runs at any PC or laptop that has a *32* or *64* bit version of Windows XP, Windows Vista or Windows 7, Windows Internet Explorer 6 or higher, a processor with a clock speed of at least 1 GHz, 512 MB of intern memory (RAM), 2 GB of free hard disk space and a graphics card which supports OpenGL. The therapy manual that contained 12 therapy sessions was handed-out in hardcopy. Illustrative screenshots of the application are online available at https://doi.org/10.4121/uuid:cacc5b31-047e-4e88-b341-cff18d76de49.

### Treatment

#### 3MR Intervention

Patients randomly assigned to the *3MR* group received a one-hour face-to-face introduction appointment with a trained therapist to be introduced to the *3MR* system and treatment manual. The treatment rationale was explained thoroughly and especially the importance of non-avoiding was discussed and highlighted. Treatment sessions were scheduled two times per week. After this introduction appointment, patients autonomously followed through the 12 sessions of *3MR* intervention at home with use of the treatment manual. At session six, the participant was called by the therapist to check progress and to see whether there were any questions or problems. The *3MR* system and an accompanying therapy manual were used to guide patients through 12 therapy sessions targeting the traumatic memories of the patients. During sessions, patients completed assignments as described in the therapy manual (abbreviated version shown here) varying from “Search for the location of this event on Google Maps, use Google Street view and answer the following questions; “Where is this?”, “What happened here?”, “What feeling does looking back at this location give you?”, to “Do you have pictures you have on your computer (add these *via* the tool “Images”) or in hard copy (add these *via* the tool “Webcam”) that date from that time period?” The upload of a picture was then followed by the questions; “Which people are on this picture?”, “Why is this picture important to illustrate your memory?”. The assignments build up to the construction of a personalized 3D environment, reflecting the actual traumatic memory of the participant with the use of corresponding 3D items. In the 3D world builder, several pre-selectable formats existed (e.g., a “desert” environment in the military version, and a “room” environment in the CSA version). Patients added items to these environments from a library, which contains hundreds of specific items such as vehicles, houses, soldiers and civilians in the military version, and coaches, tables, closets and beds in the CSA version. While looking at this personal created virtual 3D environment, patients answered classical exposure questions from the therapy manual, such as “What do you see looking at this situation?”, “What do you hear?”, “What do you smell?”. After use of the 3D world, patients were instructed to distance themselves from the memory by actively zooming out of the 3D environment before closing it.

During the *3MR* intervention the therapist could be contacted by the patient by e-mail or telephone and he or she offered feedback or help when requested. In practice, extra requests for therapist assistance were not often done and therapist assistance remained mostly confined to the mid-term contact moment initiated by the therapist at session six.

#### Treatment as usual

The control condition consisted of 12 sessions of face to face “treatment as usual” (TAU), which in this trial was a trauma-focused intervention for PTSD (i.e., Imaginal Exposure [IE], Eye Movement Desensitization and Reprocessing [EMDR], or Narrative Exposure Therapy [NET]). These approaches are applied as TAU because they are known to lead to high effect-sizes [e.g., (14)] and are recommended in guidelines from different institutions (e.g., American Psychological Association [APA], and ISTSS (14)). A total of 22 certified psychologists working at the Psychotrauma departments of PsyQ and Reinier van Arkel and the ambulatory of the Erasmus University Rotterdam were involved in TAU and/or *3MR* therapy assistance. The post measurement was conducted after 12 *3MR* or TAU therapy sessions. For ethical reasons ongoing TAU’s were not forced to stop after this point; whether or not patients had followed additional TAU during the period between the post measurement and the follow-up measurement was recorded at the three month follow-up registration.

## Data analysis

To include patients with one or more missed measurements and take into account the nested data structure of multiple measurements within a participant, a multilevel model (also known as mixed random effect or hierarchical model) was used to analyze the data. Hereby, pre, post, and 3-month follow-up measurements of the PTSD and depression symptoms are defined at Level 1 and patients at Level 2, with condition (*3MR* or TAU) as a Level 2 predictor variable [see e.g., (49); for a general overview of multilevel analysis procedures]. Hereby, a Bayesian approach was used to deal with estimation problems often encountered with small sample sizes at the higher level (49). Here, we deviated from our preregistered analysis plan (clinicaltrials.gov/ct2/show/NCT02234076) to accommodate the sample size which was lower than expected. Bayesian estimation takes both background information, the prior distribution, and the information in the data, expressed in terms of the likelihood function of the data given the parameters, into account (49). With both distributions and the use of Bayes theorem to update the prior distribution with the information in the data (50), the posterior distribution is defined for each parameter of interest. In doing so, the so-called Markov chain Monte Carlo (MCMC) method is used to estimate parameter values. The interested reader is referred to an extensive body of literature on Bayesian estimation (see e.g. Van de Schoot et al., 2014) and Bayesian multilevel modeling [see e.g. (49), (51–52)]. All models were created in Stan computational framework (http://mc-stan.org), accessed with the brms package (53) in R (R Core Team, 2020). SPSS 25 was used to generate data files from raw data files and analyze descriptive information on participants baseline differences and drop-out. Multilevel analyses were performed blinded for group allocation.

First, to assess the effects of the *3MR* condition over time, a model with random intercepts, an uninformative prior to model these intercepts, and a main effect of time was fitted to the *3MR* group for each outcome. Taking a Bayesian perspective, the outcome, e.g. PCL-5 score, is assumed to be drawn from a normal probability distribution with an unknown mean and variation parameter that is considered a random variable and described by a probability distribution (Nalborczyk et al., 2019).


$$PCL\;score_{ti}\;∼\;\mathrm{Normal}\left(\alpha_{i}+\beta time_{t},\;\sigma_{e}\right)$$

$$\alpha_{i}\;∼\;\mathrm{Normal}\left(\mu ,\;\sigma_{\alpha }\right)$$

Here, the mean PCL-5 score per patient, *i*, at a specific timepoint, *t*, is determined by a person-specific intercept and the regression coefficient for time, *β*. The person-specific intercepts, $\alpha_{i}$, are assumed to have a normal distribution with the overall mean PCL-5 score and a variance component $\sigma_{\alpha }$ (i.e. the prior distribution). For the PCL-5 scores the uninformative prior distribution had a mean of 38 and standard deviation of 25 and for the BDI-II scores a mean of 20 and standard deviation of 25, based on values reported (54). The prior distribution for the OQ-45-2 scores had a mean of 80 and a standard deviation of 50, following previous findings (48).

Subsequently, a multilevel model was fitted to each outcome including random intercepts, random slopes for the effect of time, a regression coefficient for time, for condition, and one for a cross-level interaction effect between time and condition. This means that the decrease or increase in a patient’s PCL score over time was allowed to vary across patients. As such, an additional prior distribution was specified for the person-specific slopes which were centered around the grand (i.e. mean) slope of time β. Given the novelty of *3MR,* there is no prior information on its effectiveness and its relation to TAU and an uninformative prior was specified with a mean of zero and standard deviation of 100.


$$\begin{array}{r} PCL\;score_{ti}\;∼\;\\ \;\mathrm{Normal}\left(\alpha_{i}+\beta_{i}time_{t}+\beta condition_{i}+\beta time_{t}condition_{i},\;\sigma_{e}\right) \end{array}$$

$$\alpha_{i}\;∼\;\mathrm{Normal}\left(\mu ,\;\sigma_{\alpha }\right)$$

$$\beta_{i}∼\;\mathrm{Normal}\left(\beta ,\;\sigma_{\beta }\right)$$

For the final estimates to be trustworthy, model convergence has to be obtained for each individual parameter (49). Convergence was assessed by evaluating caterpillar charts of the samples, plotting the posterior distribution, and by assessing the sensitivity of the results with respects to varying the number of chains, iterations, and burn-in phase. For those parameters not discussed here, the default prior was used to reach convergence of the models. The default priors in the brms package are chosen to be non or very weakly informative so they do not influence the results much [see (53)].

Once fitted, the parameter estimates were reported with their 95% credibility interval. This interval can be interpreted as having a 95% probability that the population value is within the limits of the interval (50). Furthermore, for the condition coefficient, histograms of the posterior samples of the slopes for condition are plotted marking the highest density interval (HDI). The HDI is a type of credibility interval that indicates the points within the interval which have a higher probability density than points outside the interval and is allowed to have unequal tails (55).

Furthermore, the dichotomous data of the M.I.N.I. Plus 5.0.0. NL were studied using descriptive statistics (diagnosis PTSD and/or current depression YES/NO) on pre, post, and follow-up measurements.

 Considering missing data due to practical hurdles in data collection; of three dropped out patients only pre diagnostic data according to the M.I.N.I. Plus 5.0.0. NL were available (which means no questionnaire data at all). In the completer group, of one participant no pre questionnaire data were available, but post questionnaire data and pre, post and follow-up M.I.N.I. Plus 5.0.0. NL data were available. Also, of one participant no post and follow-up questionnaire data were available, but pre questionnaire data and pre and post M.I.N.I. Plus 5.0.0. NL data were available. All questionnaire data were taken into account with use of the multilevel analysis. All available M.I.N.I. Plus 5.0.0. data were used in the descriptive analysis. In all other analyses the available *n* is noted when relevant. See also Figure 1 for a flow chart of participants through the study.

## Results

### Descriptive statistics

**Figure 2:**
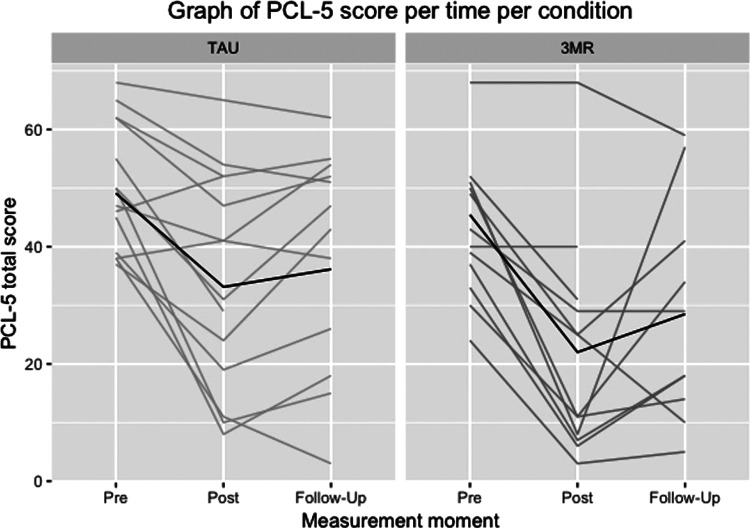
Individual patient trajectories over time for the PCL-5 in both treatment conditions, with the dark line representing the mean scores.

**Figure 3:**
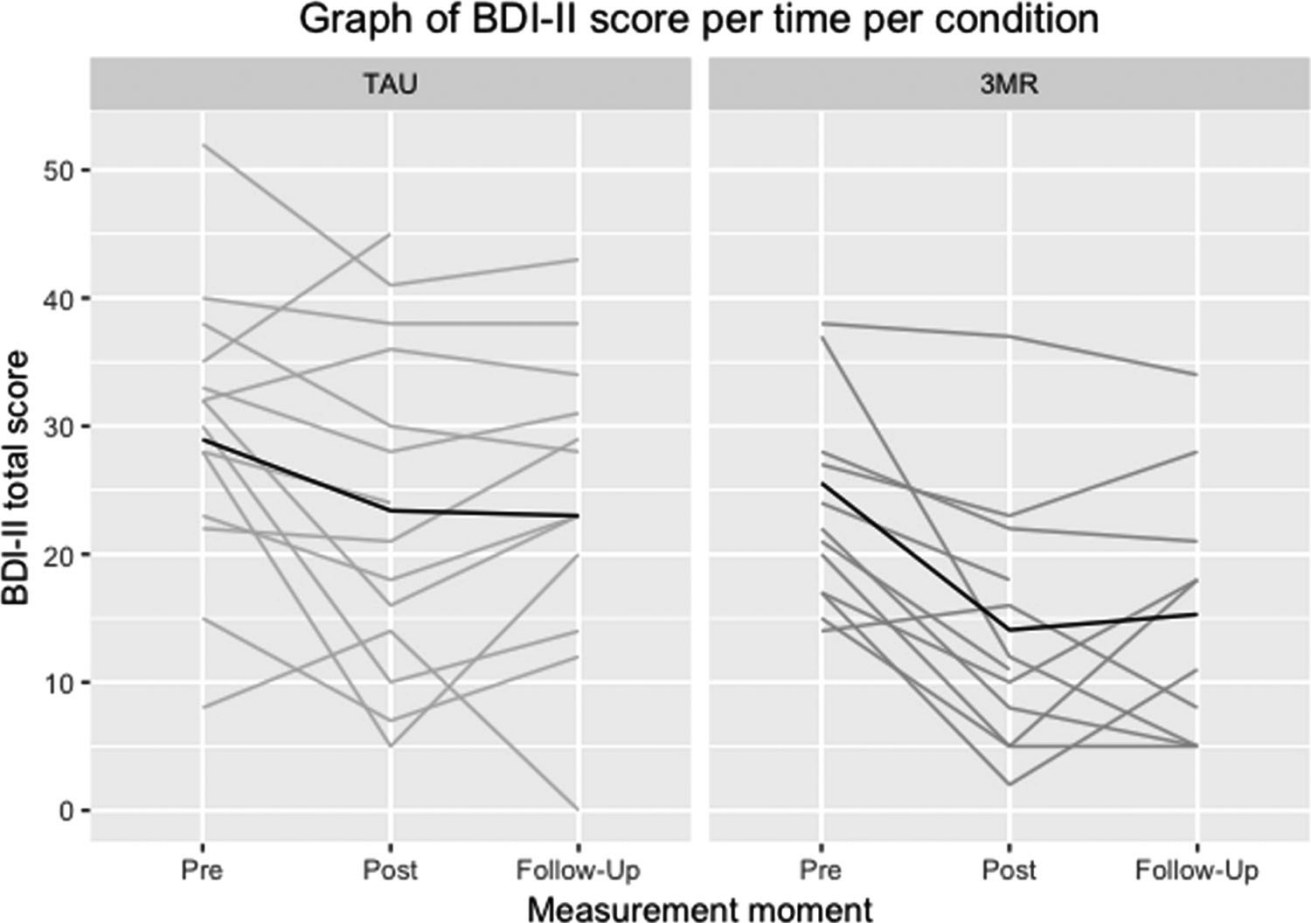
Individual patient trajectories over time for the BDI-II in both treatment conditions, with the dark line representing the mean scores.

**Figure 4:**
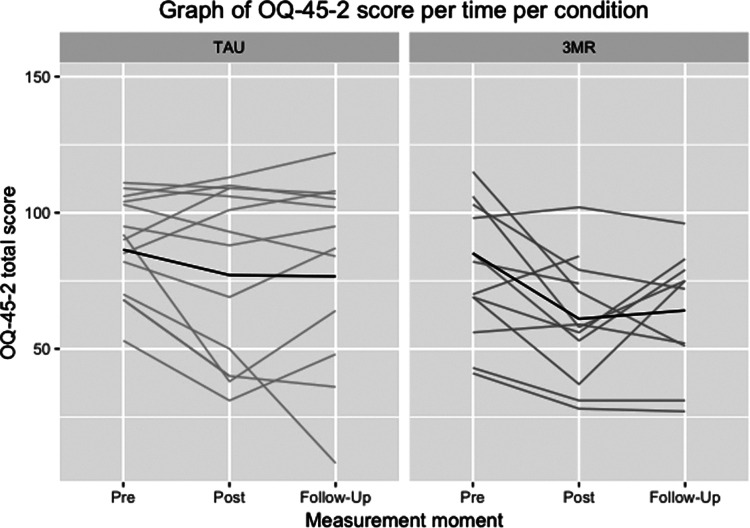
Individual patient trajectories over time for the OQ-45-2 in both treatment conditions, with the dark line representing the mean scores.

Table 3 shows the descriptive statistics of the symptom scales (PCL-5, BDI-II and OQ-45-2) in both treatment conditions (*3MR* and TAU). Figures 2–4 illustrate the individual patient trajectories over time for the three symptom scales and show there is individual variability in the symptom changes over time in both treatment conditions.

**Table 3:** Descriptives per scale per measurement moment and condition.

Scale	Time	TAU condition					*3MR* condition				
		Mean	SD	Min	Max	Missing	Mean	SD	Min	Max	Missing
PCL-5	Pre	49.10	10.22	33	68	1	45.45	13.41	18	68	
	Post	33.13	18.61	8	65	6	22	18.75	3	68	8
	Follow up	36.15	20.15	3	62	8	28.50	19.02	5	59	10
BDI-II	Pre	28.95	9.37	8	52	1	25.55	7.92	14	44	
	Post	23.4	12.56	5	45	6	14.08	9.75	2	37	8
	Follow up	23	12.82	0	43	8	15.30	10.26	5	34	10
OQ-45-2	Pre	86.4	16.56	53	111	1	85.10	25.32	14	148	
	Post	77.07	31.30	31	113	6	61	22.3	28	102	8
	Follow up	76.62	35.86	8	122	8	64.10	22.82	27	96	10

3MR, Multi Modal Memory Restructuring; TAU, Treatment as Usual; PCL-5, PTSD Checklist for the DSM 5; BDI-II-NL, Beck Depression Inventory - Second Edition; OQ-45-2, Outcome Questionnaire – 45 – Second Edition.

### M.I.N.I. Plus 5.0.0.

Descriptive statistics of the M.I.N.I. Plus 5.0.0. data show that at post measurement 81.1% of the completers in the *3MR* condition (*n* = 12) no longer met criteria for the diagnosis of PTSD, and 72.2% of the completers in the TAU condition (*n* = 18). In the *3MR* condition, 75% of the completers that met diagnostic criteria for depression at pre measurement no longer met criteria at post measurement. In the TAU condition, the rate was 50%. In both conditions one participant that did not meet diagnostic criteria at pre-measurement, did meet criteria at post-measurement. At the three month follow-up measurement, in the *3MR* condition (*n* = 11) 18.2% of the completers met criteria for PTSD and/or depression. In the TAU condition (*n* = 16) this was 25% both for PTSD and depression. In the TAU condition, two patients relapsed into diagnosis of PTSD and one into diagnosis of depression. In the *3MR* condition two patients relapsed into prior diagnosis of PTSD and one participant met criteria for depression only at the follow-up measurement.

### Drop-out analysis

Drop-out analysis revealed that drop-outs (available data *n* = 11) tended to score somewhat but not significantly different than completers (available data *n* = 29) on pre measurements of the PCL-5 [dropout *M* = 49.91, *SD* = 13.12, completers *M* = 46.28, *SD* = 11.49, *t*(38) = −0.859, *p* = 0.396], BDI-II-NL [dropout *M* = 29.19, *SD* = 6.21, completers *M* = 26.52, *SD* = 9.51, *t*(38) = −0.859, *p* = 0.396] and OQ-45-2 [dropout *M* = 94.36, *SD* = 20.76, completers *M* = 82.48, *SD* = 20.68, *t*(38) = −1.621, *p* = 0.113]. When comparing treatment conditions, the *3MR* condition suffered from a higher drop-out rate (45%) than the TAU condition (18%). This effect was noticeable but not significant *χ*^2^ (1) = 3.771; *p* = 0.104). Furthermore, slightly more patients in the CSA group dropped out than in the military group (CSA = 37.5%, military = 25%).

### Multilevel analysis

Results of the multilevel analyses are discussed per outcome measure.

#### PCL-5

A random-intercept model with a main effect of time was fitted to the data of the *3MR* condition. The results are shown in Table 4. The regression coefficients and 95% credibility intervals show that PTSD symptoms measured with the PCL-5 decreased over time with an average decrease of 22.04 points between the pre and post measurement and 14.39 between pre and follow-up measurement. Both intervals do not include zero.

**Table 4:** Posterior mean, standard error, 95% credible interval for each parameter in model predicting PCL-5 scores.

Parameter	*3MR* model			Cross-level interaction model^a^		
	Mean	SE	95%CrI	Mean	SE	95%CrI
*α*	45.52	3.82	37.9, 53.05	48.71	2.88	43.08, 54.34
β_post−pre_	−22.04	4.24	−30.43, −13.79	−16.80	3.82	−24.25, −9.27
β_follow up−pre_	−14.39	4.60	−23.42, −5.48	−12.51	4.06	−20.41, −4.50
β_condition_				−3.25	4.10	−11.29, 4.85
β_post−pre:condition_				−4.19	5.72	−15.38, 7.05
β_follow up−pre:condition_				−0.56	6.26	−12.83, 12.18
*σ*_subject_	12.96	3.24	7.23, 19.97	10.62	1.90	7.15, 14.66
σ_post−pre_				9.89	3.22	3.43, 16.41
σ_follow up−pre_				10.56	3.54	3.52,17.74
σ_e_	10.92	1.83	7.98, 15.18	7.04	1.64	3.39, 10.05

^a^ model was fitted with 3,000 warm-up samples and 8,000 iterations for 4 chains and delta was adapted to 0.99 to approach convergence.

**Figure 5:**
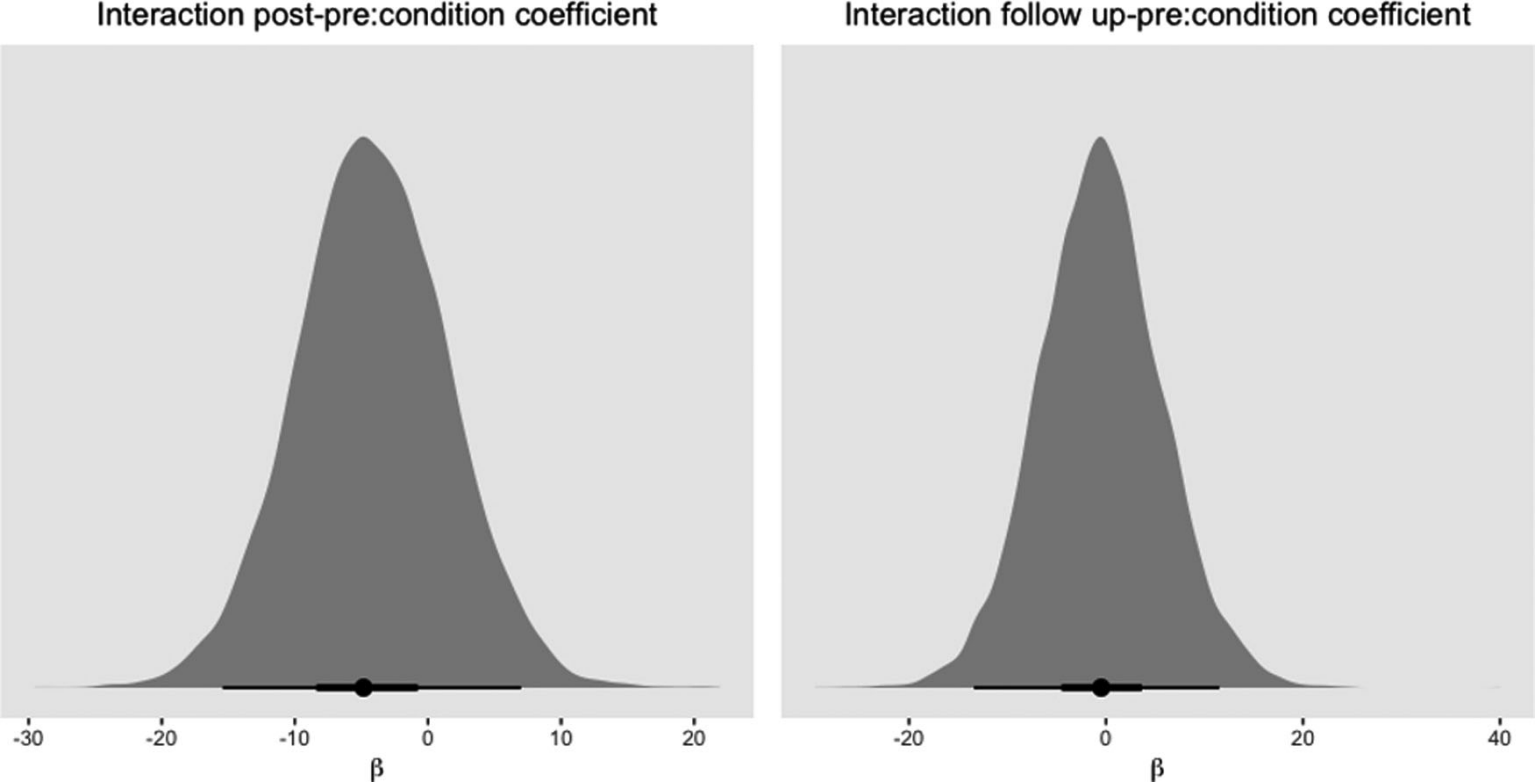
Histogram of mode of posterior distribution with 50% and 95% HDI for slopes of condition predicting PCL-5 scores. Note that a negative β coefficient indicates lower PTSD symptoms in the 3MR condition than in the TAU condition.

To compare the effects of the *3MR* and TAU condition, a model with cross-level interactions was fitted, for which the parameter estimates are also shown in Table 4. Given the many parameters and few data, reaching convergence proved to be difficult as is evident by the wide 95% credibility intervals. Overall, the 95% credibility intervals of time did not include zero indicating that PTSD symptoms decreased over time on average. This decrease in symptoms becomes slightly more uncertain over time as depicted by the wider 95% credibility interval. Appendix 3 Figure 5 shows plots of the estimates of the posterior samples of the slope for the interaction effects with the highest density intervals (HDIs). These show that the regression estimates are quite uncertain as both zero as well as positive values are included in the intervals. Still, the mode of the distribution and 50% HDI seems to be either smaller than zero or centered around zero, indicating that the treatment gains of the *3MR* intervention do at least not seem less when compared to TAU with regards to decreasing PTSD symptoms.

#### BDI-II

The parameter estimates for predicting BDI-II scores measuring depression symptoms are shown in Table 5. The model fitted to the *3MR* data shows a decrease of depression symptoms over time. On average, BDI-II scores of participants in the *3MR* condition decreased by 10.20 points [95%CrI (-15.02, −5.54)] between the pre and post measurement and −9.03 from pre to follow-up [95%CrI (-14.24, −4.21)], again excluding zero from both 95% credibility intervals.

**Table 5:** Posterior mean, standard error, 95% credible interval for each parameter in model predicting BDI-II scores.

	*3MR* model			Cross-level interaction model^a^		
Parameter	Mean	SE	95%CrI	Mean	SE	95%CrI
*α*	25.62	2.09	21.53, 29.69	28.84	2.07	24.73, 32.93
β_post−pre_	−10.20	2.37	−15.02, −5.54	−6.32	2.26	−10.83, −1.89
β_follow up−pre_	−9.03	2.55	−14.24, −4.21	−5.12	2.32	−9.65, −0.55
β_condition_				−3.24	2.95	−9.05, 2.55
β_post−pre:condition_				−3.43	3.41	−10.07, 3.32
β_follow up−pre:condition_				−3.43	3.41	−10.42, 3.38
σ_subject_	7.07	1.72	3.90, 10.91	7.75	1.29	5.39, 10.47
σ_post−pre_				4.08	2.24	0.39, 8.74
σ_follow up−pre_				3.74	2.25	0.21, 8.62
σ_e_	5.96	1.00	−14.24, 8.27	5.13	0.90	3.01, 6.77

^a^ Model was fitted with 3,000 warm-up samples and 8,000 iterations for 4 chains and delta was adapted to 0.995 and the maximum tree depth parameter to 15 to approach convergence.

**Figure 6:**
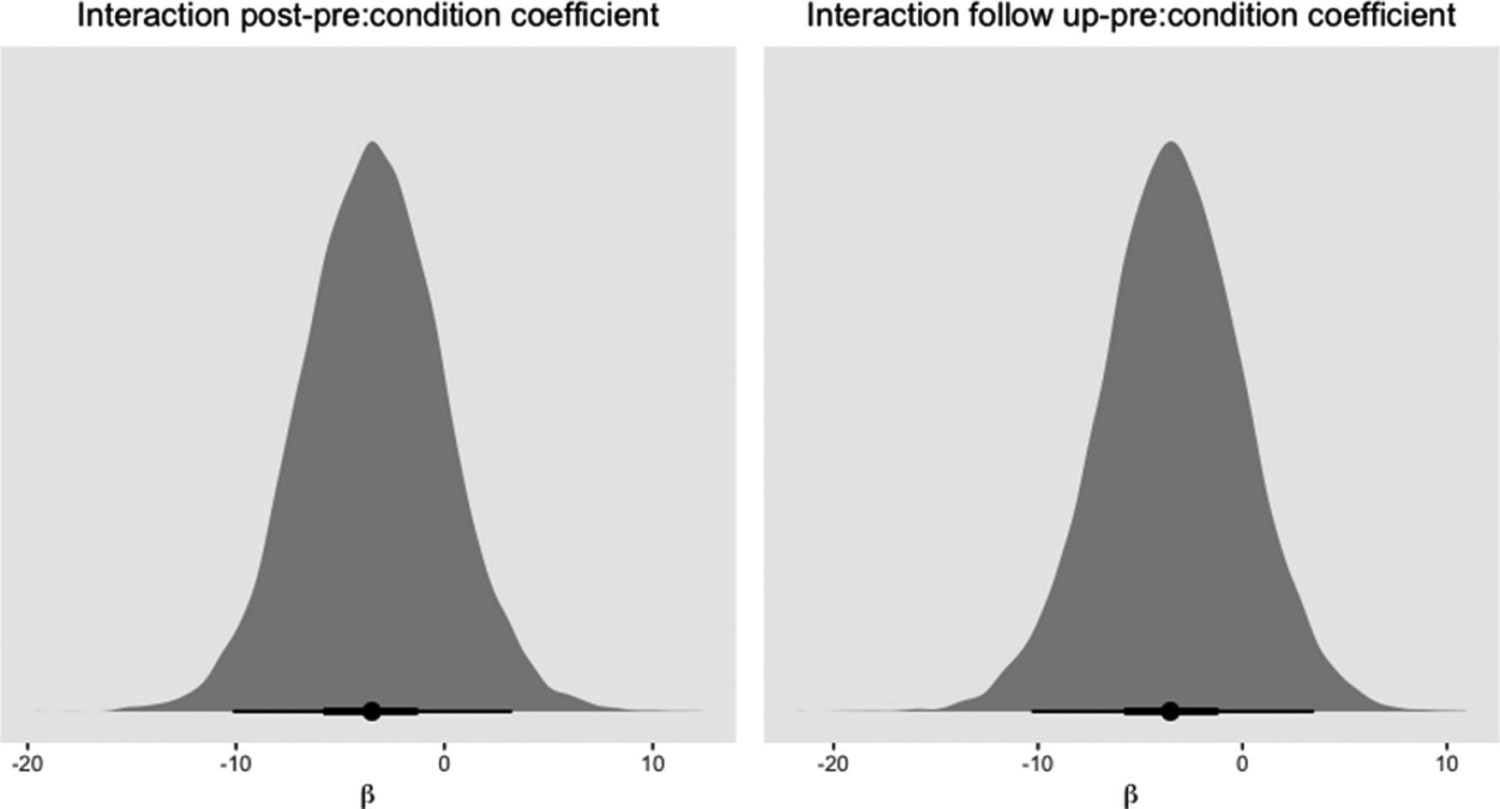
Histogram of mode of posterior distribution with 50% and 95% HDI for slopes of condition predicting BDI-II scores. Note that a negative β coefficient indicates lower depression symptoms in the 3MR group than in the TAU condition.

Further comparison of the *3MR* and TAU conditions by means of adding a cross-level interaction, shows a decrease in depression symptoms over time. This decrease becomes slightly more uncertain as time between the measurements increases, but the 95% credibility interval still does not include zero. The posterior samples plotted in Appendix 4 Figure 6 for the slopes of the interaction effects show that the mode of the distributions is negative. This implies that in most samples the decrease in depression symptoms was slightly larger in the *3MR* group compared to TAU. Also, the 50% HDI does not include zero for the interaction between condition and the pre to follow-up measurement. Although much uncertainty is still involved, this seems to suggest that the treatment gains of the *3MR* intervention with regards to decreasing depression symptoms seem not less than TAU, and sometimes even lean towards a preference for *3MR*.

#### OQ-45-2

Table 6 shows the parameter estimates for predicting overall well-being as measured with the OQ-45-2 scores. A random intercept model with a main effect of time was fitted to the data of the *3MR* condition. As indicated by the average decrease of 19.90 points from the pre to post measurement and 15.65 from pre to follow-up, and the corresponding 95% credibility intervals not containing zero, the *3MR* condition seems to lower OQ-45-2 scores over time, indicating an increase in overall well-being.

**Table 6:** Posterior mean, standard error, 95% credible interval for each parameter in model predicting OQ-45-2 scores.

	*3MR* model			Cross-level interaction model^a^		
Parameter	Mean	SE	95%CrI	Mean	SE	95%CrI
α	84.94	5.59	73.99, 95.84	85.83	5.09	75.85, 95.84
β_post−pre_	−19.90	6.12	−32.09, −8.02	−10.84	5.38	−21.45, −0.28
β_follow up−pre_	−15.65	6.68	−29.33, −2.98	−10.62	6.31	−22.98, 1.95
β_condition_				−0.75	7.22	−14.73, 13.65
β_post−pre:condition_				−7.32	8.14	−23.23, 8.81
β_follow up−pre:condition_				−2.53	9.69	−21.48, 16.59
σ_subject_	19.47	4.81	10.77, 29.69	19.55	3.26	13.64, 26.50
σ_post−pre_				10.46	5.44	0.77, 21.56
σ_follow up−pre_				14.53	6.39	2.08, 27.28
σ_e_	15.55	2.69	11.43, 21.84	11.82	2.69	5.11, 16.41

^a^ Model was fitted with 3,000 warm-up samples and 8,000 iterations for 4 chains and delta was adapted to 0.990 to approach convergence.

**Figure 7:**
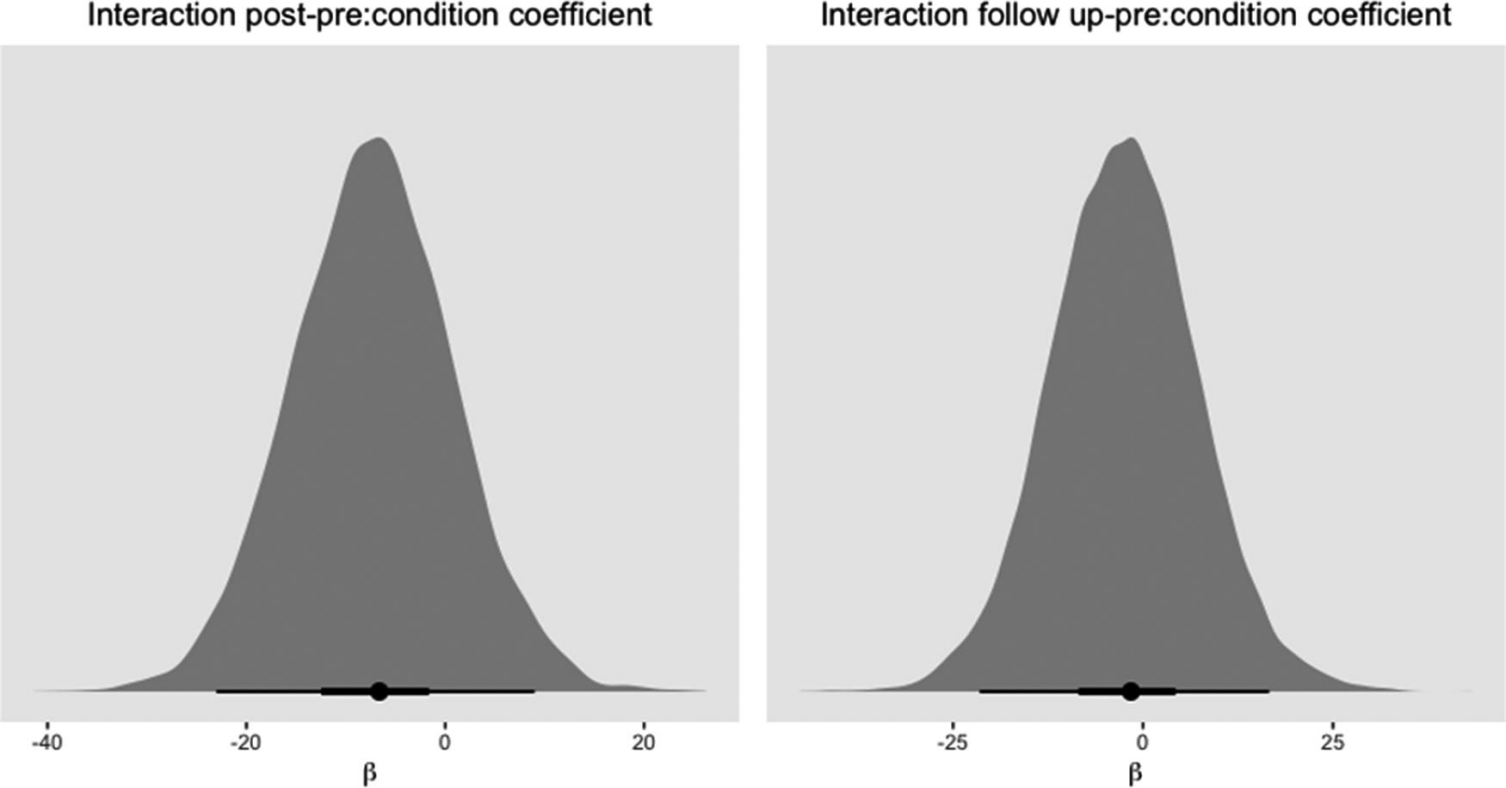
Histogram of mode of posterior distribution with 50% and 95% HDI for slopes of interaction effects predicting OQ-45-2 scores. Note that a negative β coefficient indicates lower OQ-45-2 scores in the 3MR group.

Comparing both conditions with a model including a cross-level interaction effect shows that overall OQ-45-2 scores decrease over time. With longer time periods however, the 95% credibility interval of the effect of time includes zero, suggesting less certainty that overall well-being changes over time. Similarly, the 95% credibility intervals of the interaction effects, which are on average negative, include zero. Appendix 5 Figure 7 shows plots of the estimates of the posterior samples of coefficients of the interaction effects. As shown, the mode of the distributions is negative and for the difference between the pre and follow-up measurement it is centered around zero. Again, wide HDIs show the uncertainty in the effect of time for *3MR* compared to TAU, but treatment gains seem no less than in the TAU condition.

## Discussion

This paper describes a randomized controlled trial to pilot the efficacy of a computer-based trauma intervention with elements of VR (namely patient created 3D virtual worlds viewed on a computer screen) and limited therapist assistance (*3MR* system) for the treatment of PTSD in a sample of 44 CSA and war veteran patients. The *3MR* intervention was compared to “treatment as usual” (TAU), which consisted of evidence-based trauma focused therapy such as IE, EMDR, or NET. When exploring the effects of the *3MR* intervention, results show that symptoms of both PTSD and depression seemed to decrease between pre and post measurements (after 12 sessions of respectively *3MR* or TAU). Overall well-being increased over time. When compared to TAU, no clear differences seem to emerge between the two treatment conditions when considering symptoms of PTSD, depression and overall well-being over time. This possibly indicates that the treatment gains of the *3MR* intervention are no less than those of the TAU. In addition, results of the semi-structured clinical interviews indicate that in both treatment conditions diagnoses of PTSD and depression declined from pre to post measurements. However, for example the wide credibility intervals in the results indicate that there are still large uncertainties in the effects, and that more data are needed to overcome these.

The results are in line with current literature on the efficacy of interventions for PTSD that use VR, and computer-based interventions. For example, researchers (28) describe several interventions in their meta-analysis on RCTs for the treatment of anxiety disorders (among which PTSD) and conclude that VRET is an effective and equal medium for exposure therapy. When considering the outcomes of computer-based interventions for the treatment of for example PTSD, our findings are in line with previous research which indicates that in general, computerized interventions yield comparable effect sizes as traditional psychosocial interventions in the treatment of depression and anxiety (33–36). The application of the *3MR* intervention seems promising, since the intervention was followed through autonomously by patients at home and required only limited therapist assistance. Therefore, it might not only lower expenses through decreased therapists’ costs but could also reduce travel expenses and perceived barriers for PTSD patients in remote areas or for who fear stigmatization. However, this study has several limitations. A major limitation is that it is based on a small number of patients. To allow for more conclusive results on the effects of the *3MR* intervention, additional data would be required. Furthermore, although our analysis did not show differences between drop-out rates in the *3MR* condition (45%) and the control group (18%), this percentage is high. This was rather expected since computer-based interventions generally suffer from high drop-out rates, especially when therapist involvement is low (38, 37). We expect that this low therapist involvement is also the cause of our relatively high drop-out rates. Future studies could benefit from more specific interventions to improve dropout, such as more frequent (online) contact with a therapist. Yet, high drop-out rates have also been reported in trials applying standard forms of therapy. Researchers for example report a drop-out rate of 41% of patients with CSA randomized to receive CBT, which was significantly higher than the rate of patients in the present-centered therapy and waitlist conditions (56). We further experienced more serious struggles in patient recruitment than anticipated. The sample size of the study is still small, even though patients were recruited during two years. Reasons for experienced patient recruitment difficulties might be sought for in the patients’ and therapists’ hurdles in acceptance of e-Mental Health applications for instance (57, 32). Although patients using the *3MR* intervention in this research project were generally positive in evaluating the *3MR* intervention (32), further research needs to further examine this issue. Since only the 3MR condition was computer-based, participants were not blind to the treatment condition. Consequently, expectations about the potential efficacy of a computer-based intervention might have had an impact on treatment outcome. Although we did not measure such expectations, our observation suggested that the willingness of participants to participate in one of the two conditions was equally distributed.

Lastly, trials with larger sample sizes are needed and until realized, the results of this study should be interpreted with appropriate caution. Moreover, since individual differences in treatment gain are present in this trial, further insight into the question for which patients this intervention might be specifically beneficial is needed.

In conclusion, our data seem to indicate that the *3MR* intervention may be effective in treating PTSD and its application might be especially relevant when therapist availability is low and the intention to enlarge reach of treatment efforts and improve cost-effectiveness are present. However, the small sample size and consequent large uncertainties in the estimations of the comparison between the effects of the *3MR* intervention and TAU, and high drop-out rates warrant further research.

## Data Availability

The raw data supporting the conclusions of this article will be made available by the authors, without undue reservation.

## Footnotes

^1^The informed consent of this study is available from the corresponding author on reasonable request.
